# Improved estimation of intrinsic solubility of drug-like molecules through multi-task graph transformer

**DOI:** 10.1186/s13321-025-01106-0

**Published:** 2025-10-13

**Authors:** Jiaxi Zhao, Eline Hermans, Kia Sepassi, Christophe Tistaert, Christel A. S. Bergström, Mazen Ahmad, Per Larsson

**Affiliations:** 1 Department of Pharmacy, Uppsala University, Uppsala, Sweden; 2 Pharmaceutical & Material Sciences, Johnson & Johnson, Beerse, Belgium; 3 Discovery Pharmaceutics, Johnson & Johnson, La Jolla, CA USA; 4 In-Silico Discovery, Johnson & Johnson, Beerse, Belgium

**Keywords:** Graph transformer, Muti-task learning, Quantitative structure–property relationship (QSPR), Molecular property prediction, Drug-like compounds

## Abstract

Aqueous solubility of a compound plays a crucial role throughout various stages of drug discovery and development. Despite numerous efforts using various machine learning models, accurately estimating aqueous solubility remains a challenge. One primary limitation is the absence of a single source, large dataset of druglike compounds for model training. Additionally, studies have highlighted the need for improvements in prediction algorithms and molecular representations. To address these challenges, the Johnson and Johnson (J&J) in-house solubility data was leveraged. Theoretical pH-solubility equations and in-house pKa prediction tools were utilized to calculate intrinsic solubility from J&J data. A multi-task graph transformer model was developed and trained on the calculated intrinsic solubility data of 13,306 compounds along with seven relevant physicochemical properties including solubility at pH 2/7, logP, and logD at three different pHs. When evaluated making use of high-quality test data, the developed model achieved a root mean square error (RMSE) of 0.61 and coefficient of determination (R^2^) of 0.60, demonstrating state-of-the-art performance in estimating intrinsic solubility for drug-like compounds.

**Supplementary Information:**

The online version contains supplementary material available at 10.1186/s13321-025-01106-0.

## Scientific contribution

In this study, a multi-task graph transformer model was developed to simultaneously estimate intrinsic solubility and other relevant physicochemical properties. The model achieved state-of-the art performance in estimating intrinsic solubility for drug-like compounds and demonstrated superior performance compared to single-task learning approaches and random forest models.

**Supplementary Information:**

The online version contains supplementary material available at 10.1186/s13321-025-01106-0.

## Introduction

Solubility is defined as the maximum concentration of a solute that can be dissolved in a solvent under specific temperature and pressure conditions. When the solvent is water, this solubility is referred to as aqueous solubility. Poor aqueous solubility of drug molecules can lead to incomplete absorption and suboptimal efficacy. Additionally, inadequate solubility complicates readouts and interpretation of in vitro screening assays used in drug discovery and compound profiling. Solubility experiments can be costly and time-consuming, solubility prediction tools can help identify poorly solubility drug candidates early-on, aid in the interpretation of screening assay results, and provide an opportunity to address solubility with molecular design while still in discovery.

Predicting aqueous solubility has long been a topic of significant interest, but achieving accurate predictions has consistently proven to be difficult. In 2008, Llinas et al. [1] organised the first solubility challenge. The intrinsic solubility (S_0_) of 100 drug-like compounds was provided as training data (− 8.17 to − 4.07 log molar units), entrants were then asked to predict the aqueous solubility of 32 compounds. A potentiometric method known as the ‘Chasing Equilibrium’ technique (CheqSol) was used to determine the thermodynamic solubility. The reproducible error was approximately 0.05 log molar units (abbreviated to log unit throughout the rest of the text), with later reports indicating an error of around 0.15 when comparing results between laboratories and about 0.34 when compared to the gold standard shake flask method. It was reported at that time an entire spectrum of existing methods was used by the participants. However, it was not possible to single out the ‘best model’, all methods perform equally well/badly. A second solubility competition was held after 10 years by Llinas and Avdeef [2]. Participants were asked to predict solubility with models trained on their own data and predict two test sets. High-consensus test set 1 (the ‘tight set’) contained 100 drug-like compounds taken from published literature with a standard deviation of approximately 0.17 log units, while test set 2 consisted of 32 drug-like compounds with a standard deviation of approximately 0.62 log units. Both test sets contained data generated mostly from shake-flask measurements. It was concluded that no significant improvement in predictability was made between the second and first challenge [3]. High-quality training set data, preferably from a single curated data set with at least several thousand drug-like compounds was needed to recognize significant improvements of the models and molecular representations. The two solubility challenges emphasised the importance of training and testing models on high-quality, large, drug-like datasets, while at the same time arguing that there is room for improvement in model accuracy. These challenges ignited extensive discussions around data quality, modeling approaches, and the selection of molecular descriptors. As a response to the ongoing discussion, Palmer and Mitchell [4] trained random forest models on two different solubility datasets with significant differences in the accuracy of the reported experimental values. These datasets contained the same drug-like compounds, with one having 85 compounds sourced from the CheqSol dataset in the first solubility challenge, while the other dataset compiled solubility values from literature with experimental uncertainty of 0.6–0.7 log units. They discovered the two models gave similar performance, suggesting the uncertainty in solubility measurements is not the limiting factor; rather, the algorithm and molecular representations play a more significant role.

Deep learning has emerged as a revolutionary technology, dramatically transforming various fields such as face recognition [5], language translation [6], auto driving [7], protein structure prediction [8, 9], and development of powerful large language models [10, 11]. In drug discovery, generative models [12–14], property prediction models [15–17], decision-making models [18, 19] were developed to expedite resource-intensive aspects of drug discovery, development, and clinical trials. The utilization of deep learning for solubility prediction has gained considerable attention in the last decade. Lusci et al. [20] developed undirected graph recursive neural networks (UG-RNN) and evaluated their use on various benchmark datasets such as the small Delaney data set (ESOL) [21]. The model achieved a RMSE of 0.58 on the ESOL dataset. Wiercioch et al. [22] developed a model based on an encoder-decoder structure and investigated the potential of transfer learning to enhance performance. Their approach involved pretraining the model on pKa data before fine-tuning it with aqueous solubility data from OCHEM. They found that transfer learning improved performance in data-limited scenarios, and the final model achieved a RMSE of 0.587. Francoeur et al. [23] introduced SolTranNet, a model based on the molecule attention transformer architecture that utilises SMILES as input. The model achieved an RMSE of 1.459 based on a threefold scaffold split on the AqSolDB data set. Many of these studies depend on datasets compiled from various sources, which can introduce inconsistencies due to differences in assay methodologies and lack of drug-like compounds, potentially limiting model performance and, in some cases, even presenting overly optimistic results [24]. Addressing these issues remains a critical step in improving the reliability and applicability of deep learning models in solubility prediction.

In this study, we leveraged Johnson & Johnson (J&J) in-house solubility data, which contains a large volume of solubility measurements obtained from high-throughput screening that also includes solid-state readout post solubility measurement. This dataset meets the solubility challenge requirements, i.e. the dataset contains drug-like compounds and the solubility measurements are obtained from a standard screening protocol. We incorporated theoretical solubility equations based on the Henderson-Hasselbalch equation and pKa predictions to calculate S_0_ from pH-depended solubility. A powerful multi-task graph transformer model was developed capable of predicting multiple physicochemical properties including S_0_, solubility at pH2 and pH7, logP, and logD at pH 2.6, pH7.4 and pH 10.5 simultaneously while achieving state-of-the-art performance for S_0_ prediction. Rather than limiting our approach to point predictions, we generated complete pH-solubility profiles with predicted S_0_ for compounds with multiple ionizable groups.

## Method

**Fig. 1:**
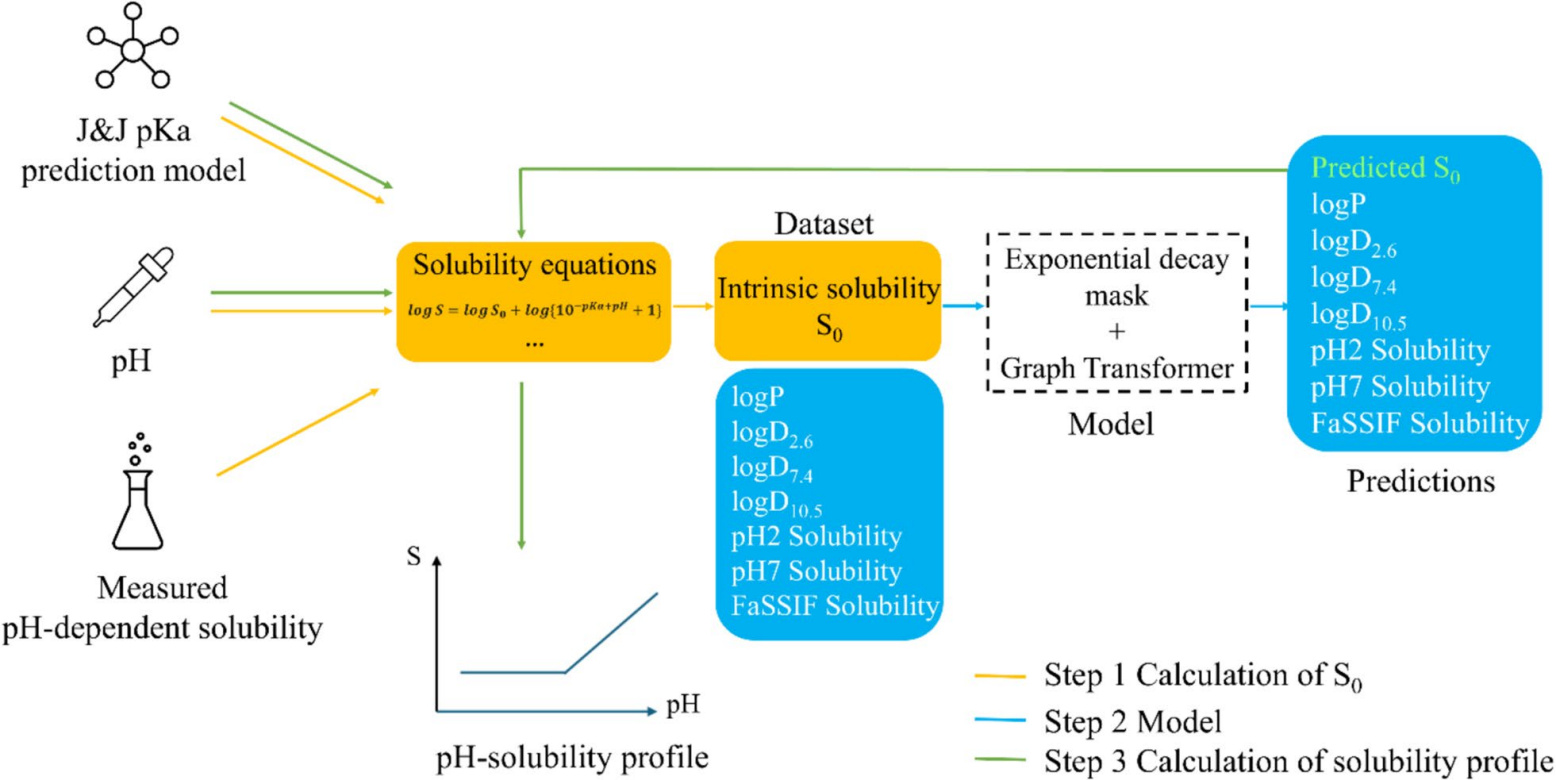
Workflow of the study. The yellow arrows represent the first step, the blue arrows represent the second step, and the green arrows represent the third step

The workflow consisted of three sequential steps (Fig. 1):

1. Calculate S_0_ from measured pH-dependent solubility and predicted pKa’s
2. Develop a multi-task graph transformer model and train it on the calculated S_0_ in order to predict S_0_
3. Derive pH-solubility profile from predicted S_0_-values and predicted pKa-values

### Step 1: generation of the S_0_ dataset

S_0_ can be estimated from the pH-solubility profile and pKa’s. In this section, a detailed breakdown of data processing for the pH-dependent solubility and pKa data is provided. Following this, an illustration is provided on how these values were utilized to calculate S_0_ and led to the compilation of a S_0_ dataset.

**pH-dependent solubility** High-throughput solubilities at pH 2 and pH 7 were generated under the same assay protocol, which included solid-state assessment of the residual solid post-solubility measurement. The assay dynamic range spanned from 0.1 to 600 µM. A filtering process was applied to only include solubility data with solid-state assessment confirming the residual solid to be crystalline. Further quality check steps to reduce the analytical variability were not conducted, as it was discovered that analytical variability in the training data will not deteriorate model performance [25]. Salt forms and duplicates were excluded. Two filtered solubility datasets were created: pH 2 solubility dataset with 12,184 data points, and pH 7 solubility dataset with a size of 14,026 data points.

**pKa** An in-house pKa prediction model was used to provide estimated pKa values for all molecules in the pH 2 and pH 7 datasets. The pKa predictions indicated that many compounds in the solubility datasets possess multiple ionizable groups. For arithmetic simplicity, we limited the scope of this study to compounds with a maximum of 3 relevant predicted pKa values. Details on the relevant pKa identification criteria can be found in the Supporting Information. This resulted in a total of 10,510 compounds in the pH 2 solubility dataset and 14,023 compounds in the pH 7 solubility dataset.

**Generate S**_**0**_ **dataset with pH-dependent solubility and pKa** The pH-dependent solubility and predicted pKa values were combined to derive S_0_ from the Eqs.  [26] summarized in Table S1. The S_0_ values derived from solubility at any pH should ideally converge to the same value, considering variability in the measured solubility values. However, for some compounds, significant differences were observed when S_0_ was derived from solubility values obtained at the two different values explored (pH 2 and pH 7). This discrepancy mainly arises when compounds are strongly ionized in either of the two conditions. In the ionized state, any slight error in pKa prediction or actual pH condition results in a large error in the calculation of S_0_. This phenomenon becomes more pronounced as the extent of ionization increases. (Additional details on the impact of pKa accuracy on S_0_ are provided in Sect. 2 of the Supporting Information) Thus, in general, it is good practice to choose the solubility at the least ionized state to calculate S_0_. Hence, we designed the following rules when S_0_ was calculated:

- For compounds with only acidic pKa values, solubility at pH 2 was used to derive S_0_
- For compounds with only basic pKa values, solubility at pH 7 was used to derive S_0_
- For compounds with both acidic and basic pKa values, if min (acidic pKa)–max (basic pKa) > 2, solubility where pH was closest to $\frac{{min\left( {acidic pKa} \right) + max\left( {basic pKa} \right)}}{2}$ (the mid-point of the smallest acidic pKa and the largest basic pKa) was chosen to calculate S_0_. Otherwise, the compounds were discarded. As a result, zwitterions were being removed from the dataset in this step.
- Solubility of compounds with no pKa values; measured solubility was directly considered as S_0_.

The selected compounds were then merged into one dataset. In case of duplicates, the difference between the S_0_ values was calculated. Only compounds with a difference of less than 0.7 log units were retained, and the smaller solubility was selected based on the assumption that it represents a more stable polymorph. Eventually, a S_0_ dataset consisting of 13,306 drug compounds was created.

**Fig. 2:**
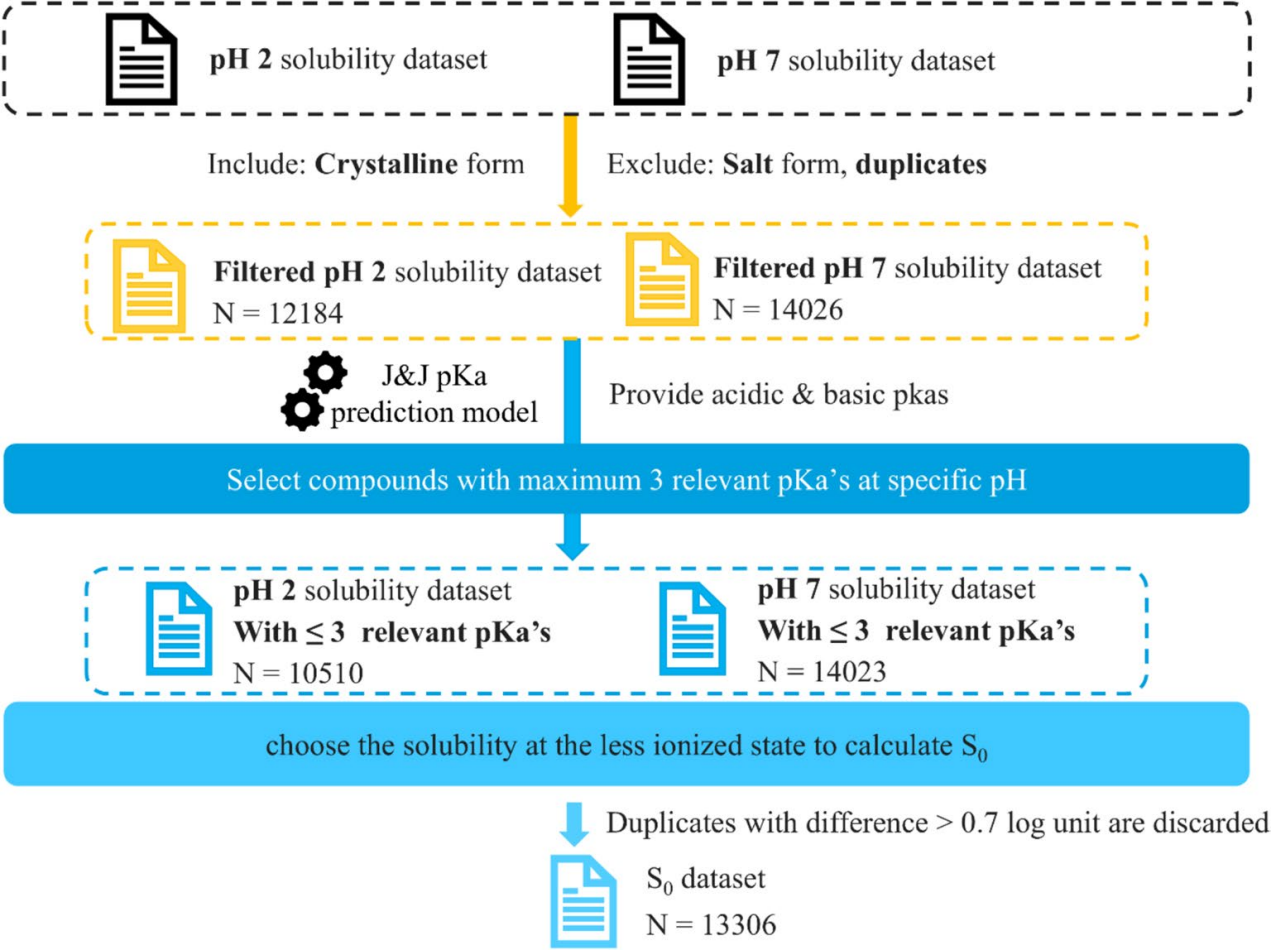
Data processing workflow for generating the S_0_ dataset

The entire data processing process is illustrated in Fig. 2.

As we wanted to develop a multi-tasking model, seven more physicochemical properties relevant to S_0_ were chosen to train the model at the same time. These properties include solubility at pH 2 and pH 7, FaSSIF solubility, logP, and logD at three different pHs. The reasoning for the selection of these related tasks is explained in the supporting information under Sect. 6. Data processing for solubility at two different pHs was already mentioned previously, FaSSIF solubility was processed with the same procedure. For logP and logD datasets, salts and duplicates were removed. Finally, datasets for all eight properties were merged to form the final dataset for model training. This dataset will be referred to as the **All-target dataset** throughout the rest of the paper.

### Step 2: model building, training and evaluation

#### Model structure

The model we developed comprised three key components: multi-task learning, an exponential decay mask as implemented in Gradformer [27], and a configuration of the graph transformer (GT) based on the GraphGPS framework [28].

GraphGPS facilitates the construction of general, powerful and scalable graph transformers [28]. It comprises three important components: positional or structural encodings that can help identify and differentiate the location of a node and overcome 1-dimensional Weisfeiler-Leman limitation; local message passing layers to capture local structure and handle local message exchange; global attention layers to allow capturing the long-range dependency. Together, the latter two components allow a balance between message-passing graph neural networks and transformer-like global attention, enabling the model to leverage the strengths of both approaches [28]. Following the framework of GraphGPS, random walk was used as positional encoding, GINE was used as the message-passing neural network.

Graph transformers tend to overlook inductive biases even though positional encoding and attention bias were designed to address this issue [27]. To overcome this problem, Liu et al. [27] introduced Gradformer, which applies an exponential decay mask to the attention matrix to integrate the intrinsic inductive bias in graph transformers. This mask is multiplied with attention score to control the decay of attention weights based on distance. This approach captures long-range information while still making use of the local information of the graph.

By utilizing the GraphGPS structure in conjunction with the exponential decay mask, the core part of the model architecture was established, which can be referred to as a feature extractor. Additionally, we wanted to harness the advantages of multi-task learning. By simultaneously learning from multiple relevant tasks, the model is able to capture general patterns across these tasks. This enhances the model’s generalizability and prediction accuracy [29]. Hence, logP, logD, two pH-dependent solubilities and FaSSIF solubility were used to train the model simultaneously with S_0_. Details of data processing were mentioned in the previous section. The model was implemented with pytorch (2.3.1) [30] and pytorch-geometics (2.5.3) [31].

#### Molecule representation

SMILES strings were transformed into graphs, where atoms were represented as nodes and bonds were represented as edges. Initial node and edge features were calculated with RDKit (2024.3.3) [32], specific node and edge features can be found in Table S2 and Table S3.

#### Model training and evaluation

**Fig. 3:**
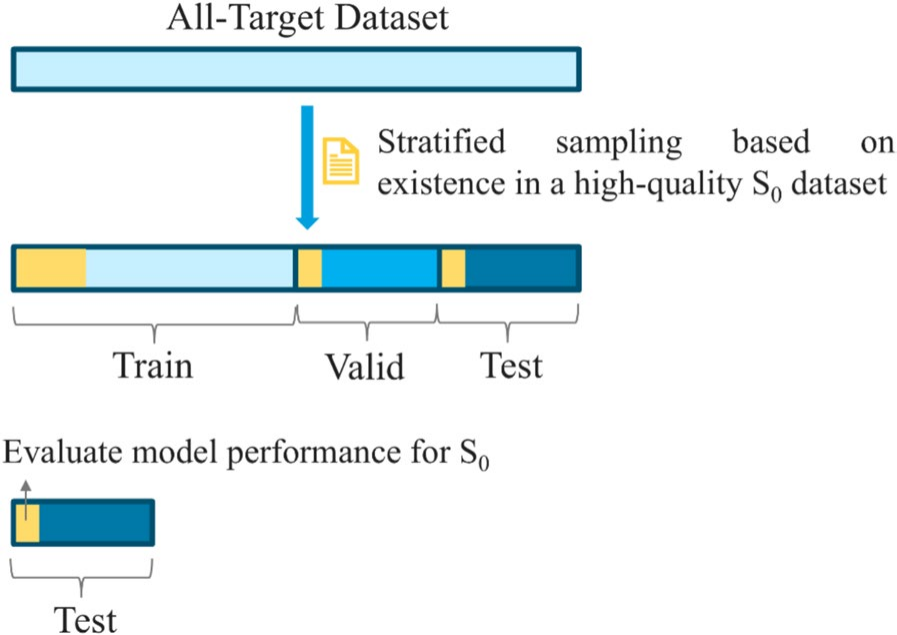
Data splitting with stratified sampling based on a high-quality S_0_ dataset. The yellow bits represent high-quality S_0_ data in each of the sub data sets

It was found by Zhao et al. [25] that models need to be evaluated on high-quality test sets in order to obtain the real performance. To achieve this, we created a high-quality S_0_ dataset from the pH 2 and pH 7 datasets following the procedures outlined here [25]. This high-quality S_0_ dataset was characterized by the inclusion of only the crystalline form of compounds and S_0_ with minimized analytical variability. The **All-target dataset** was split into train, validation, and test sets (70%, 15%, and 15% respectively) using stratified sampling based on the presence of compounds in the high-quality S_0_ dataset. In this way, high-quality S_0_ data exists among the train, validation, and test sets, making it possible to train the model on high-quality data as well as data with analytical variability (i.e. train and validation dataset), but test S_0_ prediction performance only on the high-quality S_0_ data in the test data. Figure 3 gives a graphical visualization of the process.

The model was trained on all targets simultaneously, and hyperparameter tuning was guided by the validation set. Most of the hyperparameters are the same as in the original papers [27, 28], hyperparameter tuning was only implemented on the learning rate and steps of the random walk. Optimal hyperparameter was found to be a learning rate of 0.005, and random walk steps of 20. The loss function was an uncertainty-weighted loss where uncertainty is automatically learned from the training dynamics and robust to weight initialization [33]. 200 epochs were used for training. To avoid overfitting, early stopping with steps of 30 was used. RMSE and R^2^ were used as evaluation metrics. To assess the stability of the model, five trails with different data splits were conducted, standard deviation was calculated and used for reporting model performance.

### Step 3: calculation of the pH-solubility profile

pH-Solubility profiles (pH 0–12) were calculated from predicted S_0_ and pKa’s. The relevant pKa identification step was applied to keep a maximum of 3 relevant pKa’s at the desired pH. In case more than 3 pKa’s are impacting solubility, the solubility at this pH was not determined.

To assess the performance of this calculation-prediction-calculation method, we selected compounds from the test set with measured solubilities both at pH 2 and pH 7. pH-solubility profiles were calculated for these 810 compounds based on the predicted and calculated S_0_. The two solubility points along with the two solubility profiles, are plotted together to facilitate comparison. The figure and detailed analysis are in Solubility Profile section under Results and Discussions.

## Results and discussions

### Model performance on S_0_

**Fig. 4:**
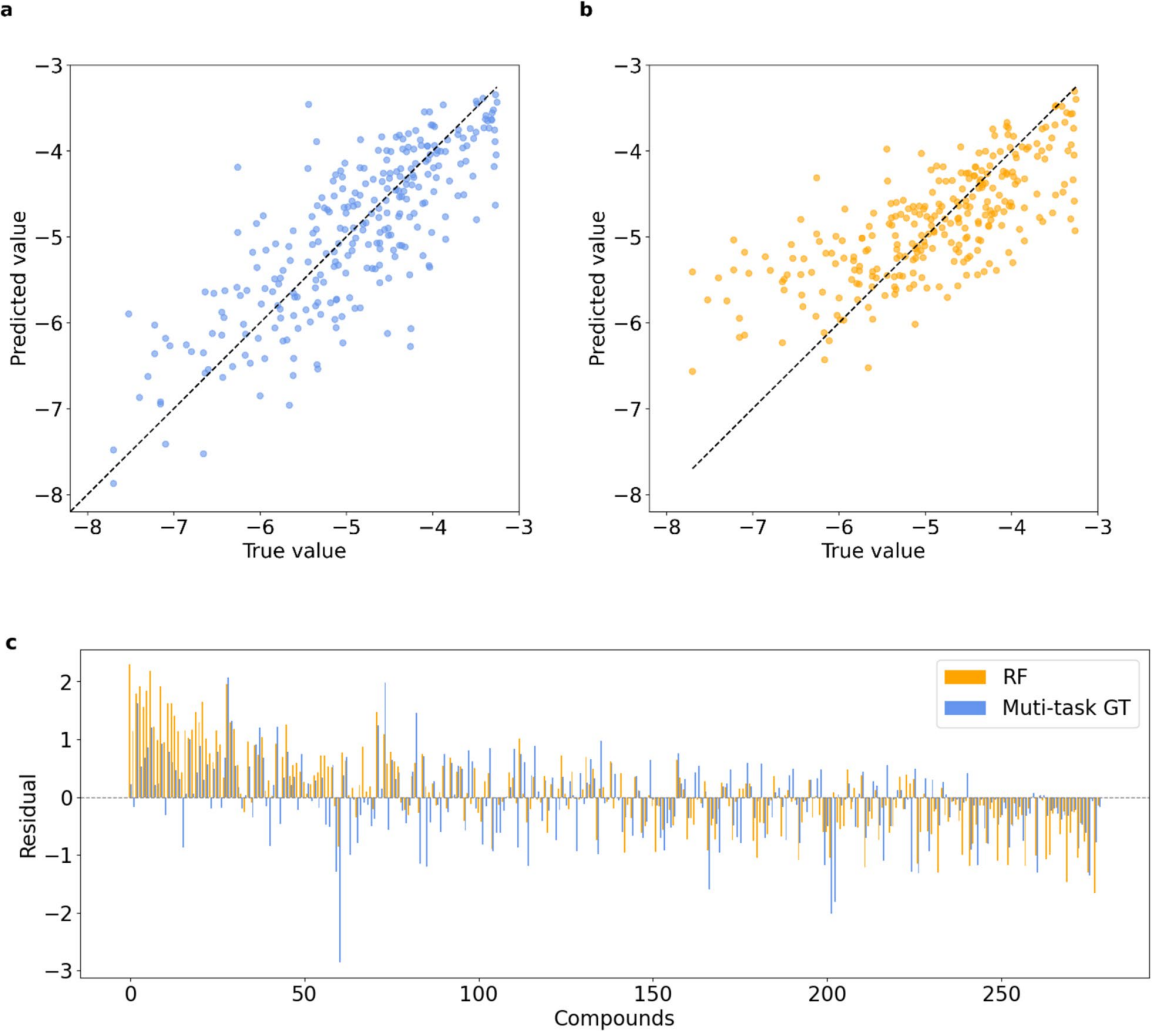
Model performance on high-quality S_0_ test data (log unit) (**a**) Predictions made by multi-task model (**b**) Predictions made by random forest (**c**) Residuals of each compound. Residuals were defined as the difference between the true value and the predicted value. Compounds were arranged along the horizontal axis in increasing order of S_0_. Vertical orange lines represent residuals calculated with predicted values made by RF and blue by multi-task GT. Vertical lines above the horizontal line indicate a positive residual and below indicate a negative residual

**Fig. 5:**
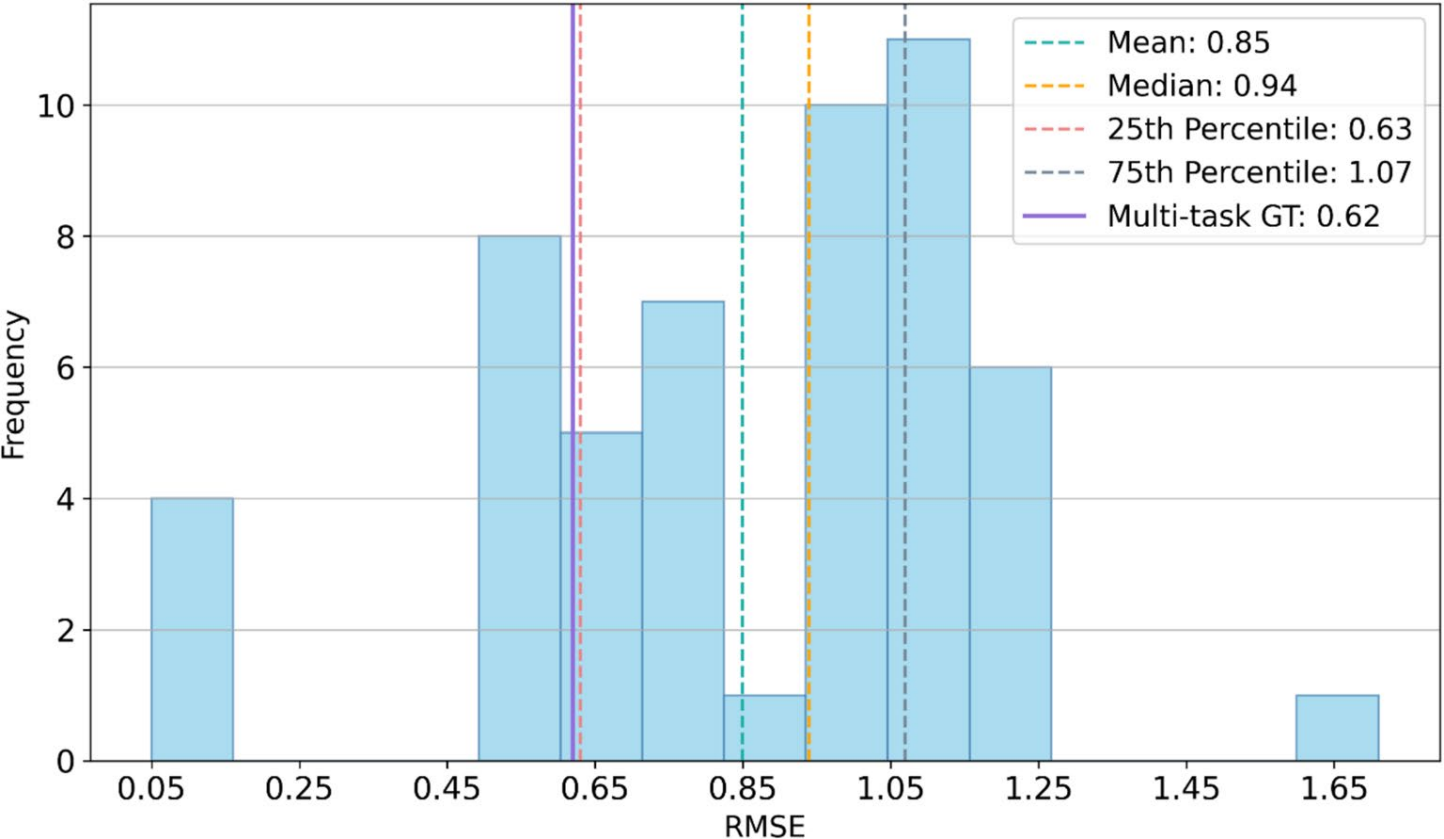
Distribution of reported thermodynamic solubility model performances from 2015 to 2023 versus performance of our Multi-task GT model

Model performance for predicting S_0_ can be found in the first column of Table 1, and correlation between predicted and measured values for one of the five trails is illustrated in Fig. 4a. With an average RMSE of 0.62 log units and R^2^ of 0.60, the developed model demonstrated strong performance in predicting S_0_. Additionally, the low standard deviation indicated that the model is stable across five different data splits. We created a histogram of reported thermodynamic solubility model performances from 2015 to 2023 (Fig. 5), based on Llompart [24] et al.’s review. Our multi-task GT model ranks among the top performers in this analysis. Notably, some of the highest-performing models in these reports were trained and evaluated on organic compounds rather than drug-like molecules. Since organic compounds are generally easier to model, they tend to yield lower RMSE values. Furthermore, some studies reported results from a single run, which may lead to overly optimistic performance estimates. Another factor contributing to inflated performance metrics is data leakage, where duplicate compounds appear in both training and test sets. In our study, we carefully curated the data, trained and evaluated drug-like molecules, and were able to confirm that the developed multi-task GT model achieves state-of-the-art performance in predicting S_0_ for drug-like compounds.

**Table 1:** Performance for muti-task model, single-task model and RF on the same refined test set

	Multi-task model	Single-task model	RF
RMSE (std)	0.62 (0.03)	0.67 (0.05)	0.71 (0.03)
R^2^ (std)	0.60 (0.05)	0.53 (0.08)	0.48 (0.03)

### Multi-task model outperforms single-task model and random forest for S_0_ predictions

The aim of this section is to determine if simultaneous training on multiple related targets enhances overall model effectiveness, and to assess whether deep learning models surpass traditional models in performance. Two more models were trained: one model based on the proposed GraphGPS and exponential decay mask architecture trained exclusively on S_0,_ and one Random Forest model (RF) that utilized ADMET predictor descriptors for S_0_ prediction. The combination of RF and ADMET predictor descriptors was identified as the optimal model by Zhao et al. [25]. The same high-quality S_0_ data was used across these three models to compare model performance. The numerical results presented in Table 1 demonstrate that the multi-task model outperforms the single-task model, with both these models exhibiting superior performance compared to RF. This indicates that training on multiple relevant targets can enhance the performance of each target compared to training on them individually. Furthermore, the results suggest that the deep learning model trained on a single large dataset outperforms classical machine learning models. From Fig. 4, it was also noticed that RF struggled to make accurate predictions for highly soluble and low soluble compounds, while our proposed deep learning model handled these predictions well.

### External test sets

The multi-task model was also tested with the first test set from the second solubility challenge (SC2). Compounds with S_0_ greater than − 3.22 log units (i.e. 600 µM) were excluded, resulting in a final test set comprising 79 compounds. Surprisingly, the performance on this external test set was poor, yielding an RMSE of 1.09. In comparison, the RF model from the previous section performed better, with an RMSE of 0.76. Thus, the developed deep learning model outperforms RF on the in-house test sets, but performed more poorly on the external test set. Similar patterns for deep learning models were observed from Francoeur et al.’s study. The SolTranNet trained on AqSolDB dataset showed much worse performance when tested on the SC2 test set or the ESOL dataset. Potential causes for this can be:

- The multi-task graph transformer model’s generalization ability is worse compared to the RF model trained with physicochemical descriptors.
- The multi-task graph transformer model is more sensitive to assay differences than the RF model.
- Different molecular representations resulted in different chemical spaces. With graph representations, the chemical space of the in-house data is different from the chemical space of the SC2 test set. In contrast, the chemical spaces defined by physicochemical properties exhibit greater similarity.

To further investigate the poor SC2 prediction performance, the chemical spaces based on latent features obtained from the feature extractor for both J&J intrinsic solubility and SC2 datasets are plotted in Figure S7. It can be observed from the plot that the poorly predicted compounds were mostly located in the sparsely populated regions of the J&J training data, suggesting that limited representation learned in these regions may contribute to the larger prediction errors. A few poorly predicted SC2 compounds sat in dense regions, which could indicate our multi-task graph transformer model is sensitive to assay shift (assay differences).

Additionally, single-task training was conducted on the ESOL dataset [21] (1115 compounds after removing duplicates), with 80% of training data and 20% of test data. The single-task model achieved an RMSE of 0.60 with SD of 0.02 on the test set and RMSE of 1.03 on the SC2 test set. An RF model was trained with ADMET predictor descriptors on the same ESOL data, which resulted in an RMSE of 0.60 with SD of 0.04 on the test set, and RMSE of 0.87 on the test set. The two models exhibited similar performances on the ESOL test set based on the average RMSE across five splits of the data. However, with a lower SD, the single-task model was more stable. Similar performance may suggest that the model is constrained by the small size of the dataset and that the dataset itself is relatively easier for the models to learn and achieve equally good performances, since the compounds do not extensively represent the drug-like chemical space. Although the RF model performed well on the ESOL test set, its predictions on the SC2 test set (RMSE of 0.87) were worse than the RF model trained on the J&J data (RMSE of 0.76). This discrepancy indicates that the applicability domains of the two datasets differ significantly, with one encompassing the drug-like chemical space while the other does not.

### pH-solubility profile

**Fig. 6:**
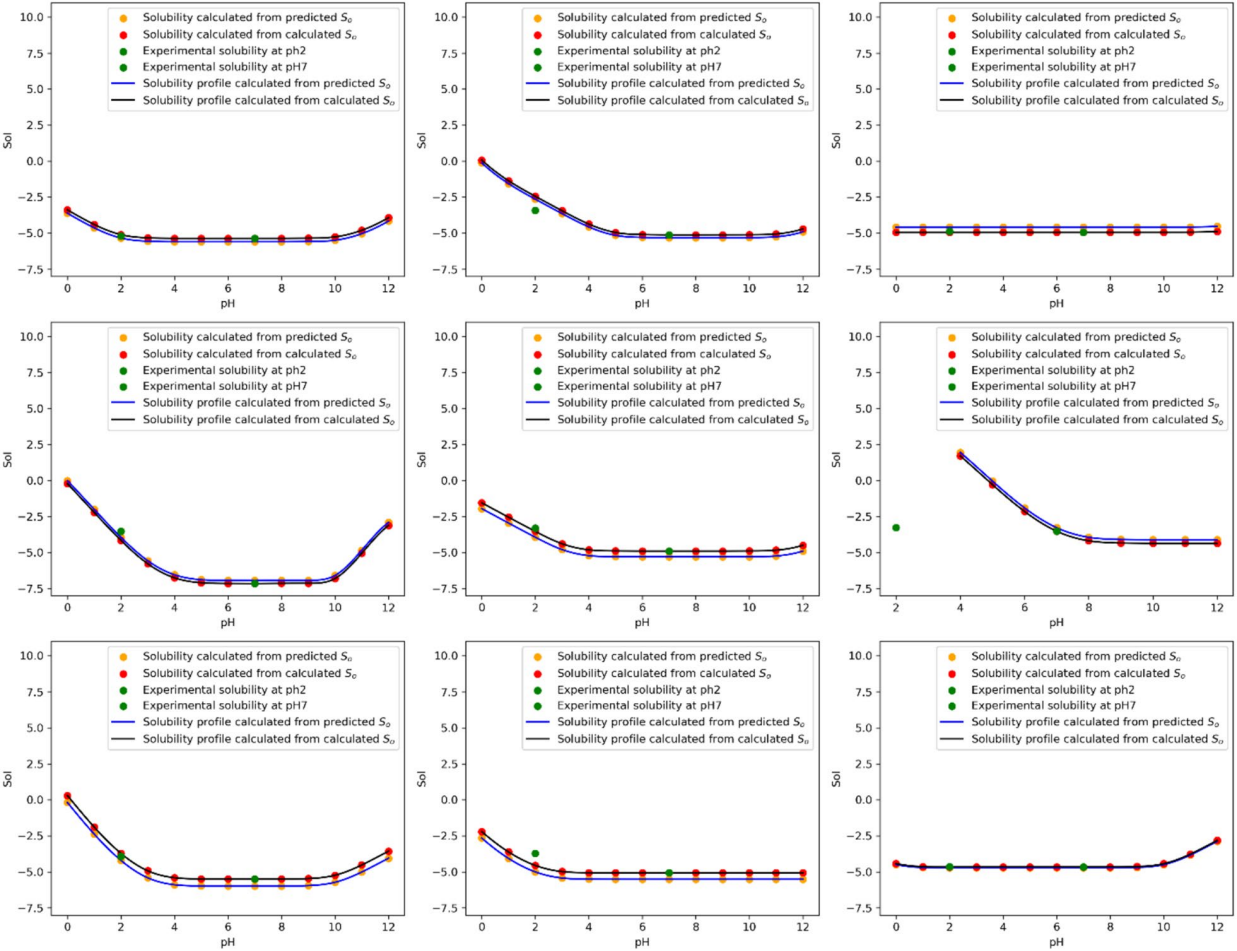
pH-Solubility profiles for nine compounds

To assess the effectiveness of the method in generating pH-solubility profiles, the calculated pH-solubility profiles from the calculated S_0_ are compared with the predicted S_0_, along with the two solubility points. Figure 6 showcases nine representative examples. The plots reveal that the difference between the two solubility profiles across the pH range of 0–12 remains nearly constant, equal to the S_0_ prediction error. In other words, the S_0_ prediction error is consistently reflected in the solubility curve calculations. This is expected, based on the mathematical equations used for the pH solubility profile.

For the 810 compounds in the test set with solubilities at pH 2 and pH 7, the S_0_ predictions yielded an average RMSE of 0.51 log units, with 75% of the compounds exhibiting errors smaller than 0.71. These results demonstrate that our method provides relatively accurate predictions for solubility profiles.

Although most of the time the two measured solubility data points align with the solubility curve derived from the calculated S_0_, there are instances where one of the points does not fall on the curve. As mentioned above, only 1 of the measurements was used to calculate S_0_ and discrepancies may arise from measurement errors in solubility or inaccuracies in pKa predictions.

## Conclusion

Given the large size, consistency in assay conditions, and druglike chemical space, the solubility database utilized in this investigation provides a valuable opportunity for assessing model performance on high-quality data. The pH-solubility equations were used to derive S_0_ from pH 2 and pH 7 solubility measurements and predicted pKa’s. A multi-task graph transformer model was trained on the S_0_ values along with seven other relevant physicochemical properties: solubility at pH 2 and pH 7, logP, logD at three different pHs. The model achieved state-of-the-art performance in predicting S_0_ for druglike compounds. Comparative analyses revealed that multi-task learning outperformed single-task learning approaches and RF methods. Instead of providing only point predictions, with the predicted S_0_ and pKa’s we generated a reliable solubility-pH profile. A critical factor for the success of this method is the accuracy of pKa predictions or measurements.

## Supplementary Information

Additional file 1 (The supporting information document includes pH-solubility equations used; The influence of pKa accuracy on S0 calculation; Details of relevant pKa identification step; Distribution of datasets; Dataset properties; Correlation of logP, logD and solubility; Molecular representation; Model parameters; Model performance on all eight physicochemical properties; Analysis and reasoning for SC2 data prediction performance.)

## Data Availability

Source codes, SC2 dataset and ESOL dataset are available on the GitHub repository https://github.com/JXZhaocc/Muti-task-GT-sol. The J&J datasets remain undisclosed for confidentiality reasons.

## Abbreviations

CheqSol: ‘Chasing Equilibrium’ Technique
ESOL: Small Delaney Data Set
J&J: Johnson and Johnson
R^2^: Coefficient of Determination
RF: Random Forest
RMSE: Root Mean Square Error
S_0_: Intrinsic Solubility
