# Supplementary material for: Improved estimation of intrinsic solubility of drug-like molecules through multi-task graph transformer
Source: J Cheminform. 2025 Oct 13;17:153. doi: 10.1186/s13321-025-01106-0 (PMC12516829; doi:10.1186/s13321-025-01106-0)
Supplement: Supplementary file 1 — Additional file 1 (The supporting information document includes pH-solubility equations used; The influence of pKa accuracy on S0 calculation; Details of relevant pKa identification step; Distribution of datasets; Dataset properties; Correlation of logP, logD and solubility; Molecular representation; Model parameters; Model performance on all eight physicochemical properties; Analysis and reasoning for SC2 data prediction performance.) [file 13321_2025_1106_MOESM1_ESM.pdf]

Supporting information

Improved Estimation of Intrinsic Solubility of Drug-like Molecules through Multi-task Graph Transformer

Jiaxi Zhao[1], Eline Hermans[2], Kia Sepassi[3], Christophe Tistaert[2], Christel A. S. Bergström[1], Mazen Ahmad[4], Per Larsson[1*]

[1]Department of Pharmacy, Uppsala University, Sweden [2] Pharmaceutical & Material Sciences, Johnson & Johnson, Beerse, Belgium [3]Discovery Pharmaceutics, Johnson & Johnson, La Jolla, CA, USA [4] In-silico discovery, Johnson & Johnson, Beerse, Belgium

Contents

1. pH-solubility equations for Mono-, Di-, and Triprotic Molecules.................................... 3
2. The influence of pKa accuracy on S_0 calculation 4
3. Relevant pKa identification 5
4. Distribution of the datasets 6
5. Dataset properties... 6
6. Correlation of logP, logD and solubility 9
7. Molecular representation 10
8. Model parameters... 10
9. Model performance 11
10. Analysis and reasoning for SC2 data prediction performance.................................... 11

1. pH-solubility equations for Mono-, Di-, and Triprotic Molecules

pH-Solubility equations for mono-, Di-, and Triprotic Molecules can be found in Table S1.

Table S1. Solubility-pH Equations for mono-, Di-, and Triprotic Molecules

Equilibrium Ionization	Equilibrium Solubility	Solubility Equation
$HA \overset{K_a}{\leftrightarrow} H^+ + A^-$	$HA_{(S)} \overset{S_0}{\leftrightarrow} HA$	$\log S = \log S_0 + \log\{10^{-pK_a+pH} + 1\}$
$HA^- \overset{K_{a2}}{\longleftrightarrow} H^+ + A^{2-}$<br>$H_2A \overset{K_{a1}}{\longleftrightarrow} H^+ + HA^-$	$H_2A_{(S)} \overset{S_0}{\leftrightarrow} H_2A$	$\log S = \log S_0 + \log\{10^{-pK_{a2}-pK_{a1}+2pH} + 10^{-pK_{a1}+pH} + 1\}$
$HA^{2-} \overset{K_{a3}}{\longleftrightarrow} H^+ + A^{3-}$<br>$H_2A \overset{K_{a2}}{\longleftrightarrow} H^+ + HA^{2-}$<br>$H_3A \overset{K_{a1}}{\longleftrightarrow} H^+ + H_2A^-$	$H_3A_{(S)} \overset{S_0}{\leftrightarrow} H_3A$	$\log S = \log S_0 + \log\{10^{-pK_{a3}-pK_{a2}-pK_{a1}+3pH} + 10^{-pK_{a2}-pK_{a1}+2pH} + 10^{-pK_{a1}+pH} + 1\}$
$BH^+ \overset{K_a}{\leftrightarrow} H^+ + B$	$B_{(S)} \overset{S_0}{\leftrightarrow} B$	$\log S = \log S_0 + \log\{10^{+pK_a-pH} + 1\}$
$BH^+ \overset{K_{a2}}{\longleftrightarrow} H^+ + B$<br>$BH_2^{2+} \overset{K_{a1}}{\longleftrightarrow} H^+ + BH^+$	$B_{(S)} \overset{S_0}{\leftrightarrow} B$	$\log S = \log S_0 + \log\{10^{+pK_{a2}+pK_{a1}-2pH} + 10^{+pK_{a2}-pH} + 1\}$
$BH^+ \overset{K_{a3}}{\longleftrightarrow} H^+ + B$<br>$BH_2^{2+} \overset{K_{a2}}{\longleftrightarrow} H^+ + BH^+$<br>$BH_3^{3+} \overset{K_{a1}}{\longleftrightarrow} H^+ + BH_2^{2+}$	$B_{(S)} \overset{S_0}{\leftrightarrow} B$	$\log S = \log S_0 + \log\{10^{+pK_{a3}+pK_{a2}+pK_{a1}-3pH} + 10^{+pK_{a3}+pK_{a2}-2pH} + 10^{+pK_{a3}-pH} + 1\}$
$HX \overset{K_{a2}}{\longleftrightarrow} H^+ + X^-$<br>$H_2X^+ \overset{K_{a1}}{\longleftrightarrow} H^+ + HX$	$HX_{(S)} \overset{S_0}{\leftrightarrow} HX$	$\log S = \log S_0 + \log\{10^{+pK_{a1}-pH} + 10^{-pK_{a2}+pH} + 1\}$
$HX \overset{K_{a3}}{\longleftrightarrow} H^+ + X^-$<br>$H_2X^+ \overset{K_{a2}}{\longleftrightarrow} H^+ + HX$<br>$H_3X^{2+} \overset{K_{a1}}{\longleftrightarrow} H^+ + H_2X^+$	$HX_{(S)} \overset{S_0}{\leftrightarrow} HX$	$\log S = \log S_0 + \log\{10^{+pK_{a2}+pK_{a1}-2pH} + 10^{+pK_{a2}-pH} + 10^{-pK_{a3}+pH} + 1\}$
$HX^- \overset{K_{a3}}{\longleftrightarrow} H^+ + X^{2-}$<br>$H_2X \overset{K_{a2}}{\longleftrightarrow} H^+ + HX^-$<br>$H_3X^+ \overset{K_{a1}}{\longleftrightarrow} H^+ + H_2X$	$H_2X_{(S)} \overset{S_0}{\leftrightarrow} H_2X$	$\log S = \log S_0 + \log\{10^{-pK_{a3}-pK_{a2}+2pH} + 10^{-pK_{a2}+pH} + 10^{-pK_{a1}-pH} + 1\}$

2. The influence of pKa accuracy on S_0 calculation

Starting from S_0 for a monoprotic acid:

$$S = S_0[1 + 10^{(pH-pKa)}]$$

For a monoprotic base:

$$S = S_0[1 + 10^{(pKa-pH)}]$$

For convenience, put:

$$S = S_0[1 + K], \text{ with } K = 10^{\sigma(pH-pKa)} \ (\sigma = 1 \text{ for acids and } -1 \text{ for bases}))$$

Assume the error in predicted pKa is Δ (pKa* = pKa + Δ, with pKa* = predicted pKa)

S_0 calculated from S and a predicted pKa:

$$S_0^* = \frac{S}{[1+K^*]}, \text{ with } K^* = 10^{\sigma(pH-pKa^*)}$$

S_0 calculated from S and a measured pKa:

$$S_0 = \frac{S}{[1+K]}$$

The error can be expressed as the ratio between S_0 * and S_0:

$$\frac{S_0^*}{S_0} = \frac{[1+K]}{[1+K^*]}, \text{ which becomes } \frac{S_0^*}{S_0} = \frac{[1+10^{\sigma(pH-pKa)}]}{[1+10^{\sigma(pH-pKa^*)}]}$$

$$\frac{S_0^*}{S_0} = \frac{[1+10^{\sigma(pH-pKa)}]}{[1+10^{\sigma(pH-pKa-\Delta)}]}$$

$$\frac{S_0^*}{S_0} = \frac{[1+K]}{[1+10^{-\sigma\Delta}K]}$$

This expression links the changes in S_0* with errors in predicted pKa. Figure S1 illustrates the effect of a 1 log unit error in pKa prediction (Δ = 1) on S_0* for monoprotic acid and monoprotic base, as a function of the relative position of pH to pKa. X axis represents $K = 10^{\sigma(pH-pKa)}$, y axis represents the ratio $\frac{S_0^*}{S_0}$. For acids, the green error curve shows a sigmoidal increase: Starting near 1, the error ratio rises sharply around $K = 10^0$ (where $\frac{S_0^*}{S_0} \approx 1.81$) and approaches a plateau near 10.

For bases, the orange curve also starts near 1 but gradually decreases around K = 10^0 (where $\frac{S_0^*}{S_0} \approx$ 0.92) and reaching a plateau near 0. Both curves demonstrate that even an error of 1 log unit in pKa prediction can introduce substantial deviations in the calculated S_0*.

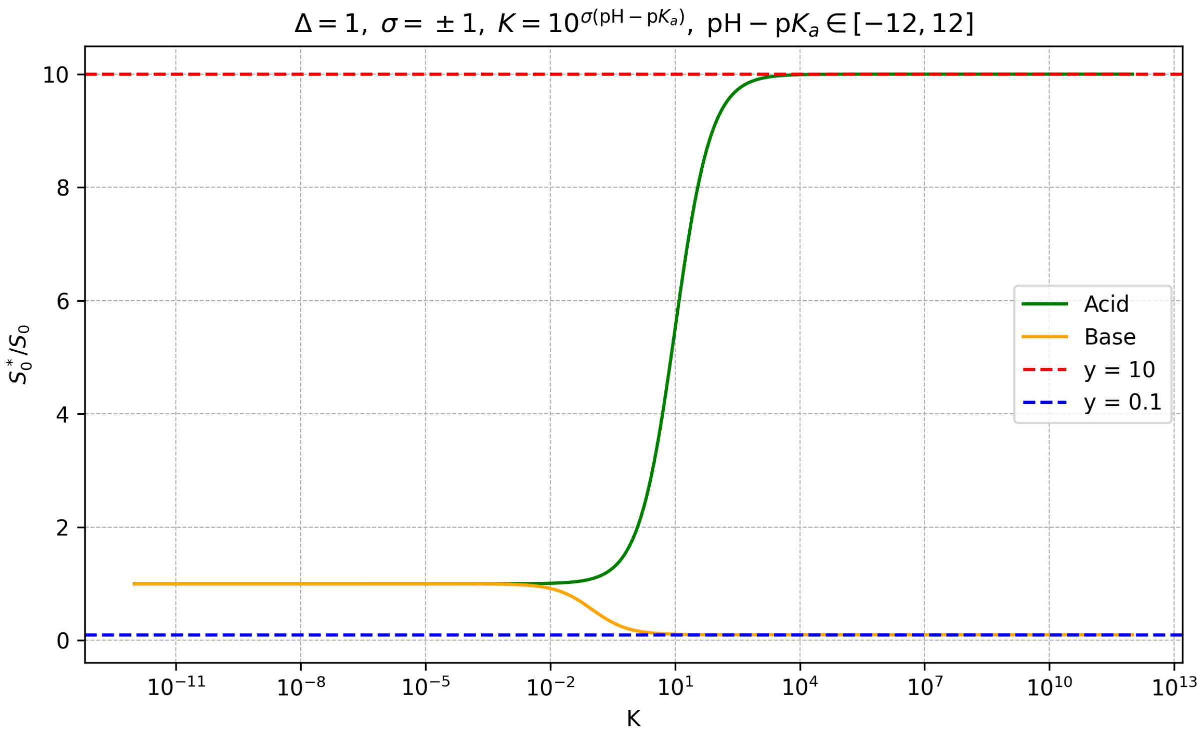

Figure 1S. $\frac{S_0^*}{S_0}$ as a function of K = $10^{\sigma(pH-pKa)}$ when Δ=1 and σ=±1. The green and orange curves represent monoprotic acid and monoprotic base, respectively. The red and blue dashed lines indicate the theoretical asymptotes at 10 and 0.1, respectively.

3. Relevant pKa identification

The criteria for relevant pKa identification at a specific pH are as follows:

- For acidic pKas, pKa that satisfies pKa < pH - 2 is considered relevant pKa
- For basic pKas, pKa that satisfies pKa > pH + 2 is considered relevant pKa

In the cases of pH 2 and pH 7:

- For compounds in the pH 2 solubility dataset, basic pKa > 0 and acidic pKa < 4 are selected
- For compounds in the pH 7 solubility dataset, basic pKa > 5 and acidic pKa < 9 are selected

Only relevant pKa's are used to calculate intrinsic solubility with the pH-solubility equations. Compounds with more than 3 relevant pKa's impacting solubility at the specific pH were removed.

4. Distribution of the datasets

Distribution of the 8 data sets are illustrated in Figure S2. All solubility data sets are right skewed. Solubility at pH2, pH 7 and FaSSIF solubility fall within the range of -8 to -3, while the intrinsic solubility dataset also have values lower than -8. logP and logD at ph7.4 display roughly normal distributions, whereas logD at pH 2.6 and logD at pH 10.5 are slightly right skewed. Notably, approximately one-third of the compounds are classified as 'grease balls' (logD >3).

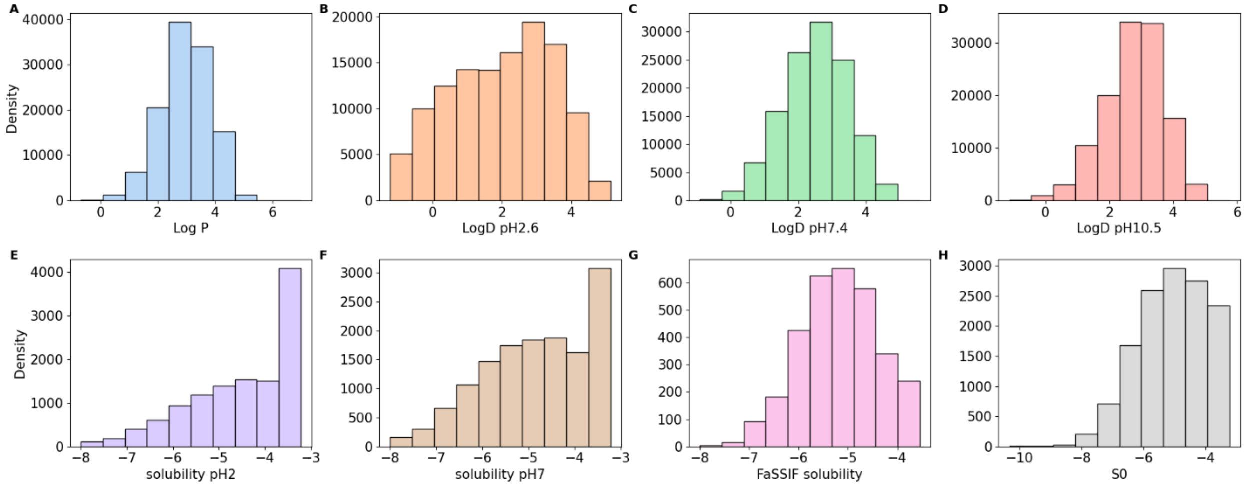

Figure S2. Distribution of eight data sets

5. Dataset properties

The chemical spaces based on ECFP fingerprints for J&J intrinsic solubility and ESOL datasets were visualized with UMAP in Figure S3(a). The red and blue dots represent compounds from the ESOL and J&J datasets, respectively. The blue dots cover a significantly larger region, indicating that the J&J dataset spans a broader chemical space and exhibits greater structural diversity. Figure S3(b) shows the chemical spaces as defined by physicochemical properties for both datasets. J&J dataset exhibited a broader and more dispersed distribution, indicating diverse physicochemical profiles, whereas ESOL dataset occupied a narrower region. While the two chemical spaces partially overlap, the two datasets also span distinct and separate regions.

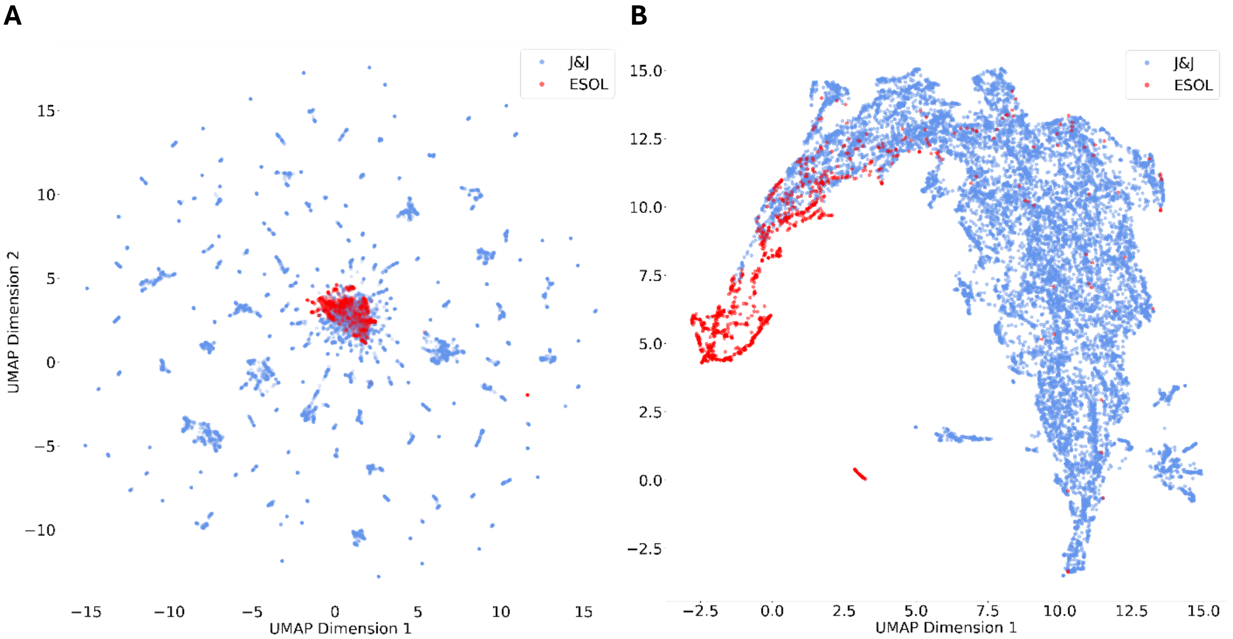

Figure S3. Chemical space of J&J and ESOL datasets based on (a) ECFP fingerprints and (b) physicochemical properties. J&J dataset in blue and ESOL in red.

Distributions of several physicochemical properties for the J&J and ESOL datasets are shown in Figure S4. Clear distribution differences between the two datasets can be observed for each of these properties.

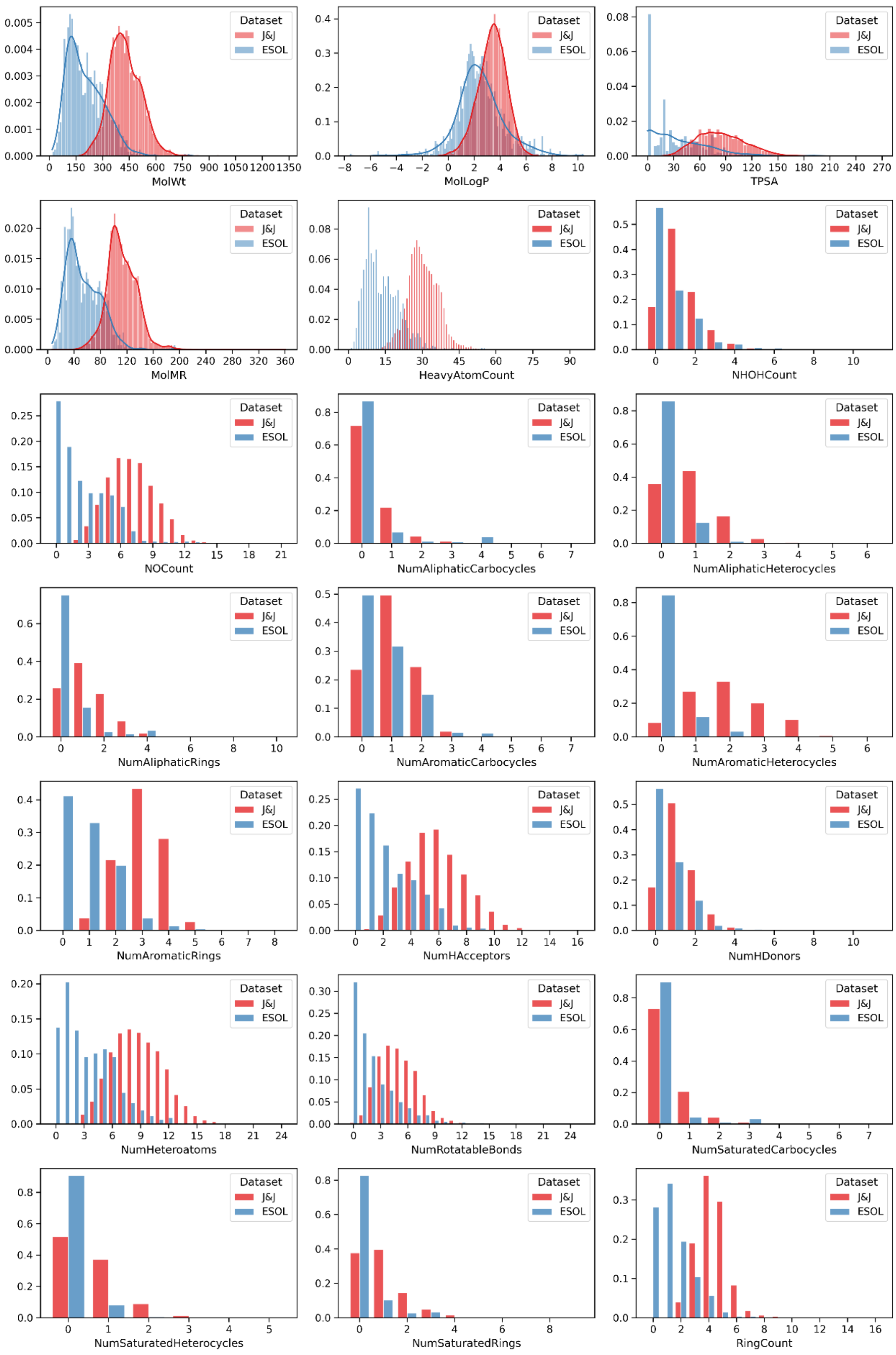

Figure S4. Physicochemical properties of J&J and ESOL dataset. J&J in red, and ESOL in blue.

6. Correlation of logP, logD and solubility

By simultaneously training on several related targets, multi-task learning can extract shared features from these tasks, thereby improving the generalizability of the model and enhancing the prediction accuracy for each task (target). In this study, we trained on eight related targets: intrinsic solubility, solubility at pH2, solubility at pH7, logP, and logD at pH2.6, pH 7.4 and pH 10.5. The relationships between solubility and logD can be categorized into three groups:

- For compounds that are neutral within pH2-12, solubility at pH 2 and pH 7 are the same and equal to the intrinsic solubility, the logD values at pH 2.6, pH 7.4, and pH 10.5 are also equal to each other.
- For ionizable compounds: acidic compounds have lower solubility at pH 2 than at pH 7, and logD at pH 2.6 is higher than at pH 7.4. Conversely, basic compounds exhibit higher solubility at pH 2 than at pH 7, with logD at pH 7 greater than at pH 2.6.

Figure S5 shows a graphical illustration of the negative correlation between logP and intrinsic solubility: compounds with low intrinsic solubility tend to have high logP values, and vice versa.

Compounds with high intrinsic solubility tend to have higher FaSSIF solubility. The presence of bile salts and other surfactants in FaSSIF can enhance the solubility of lipophilic or poorly soluble drugs beyond their intrinsic water solubility.

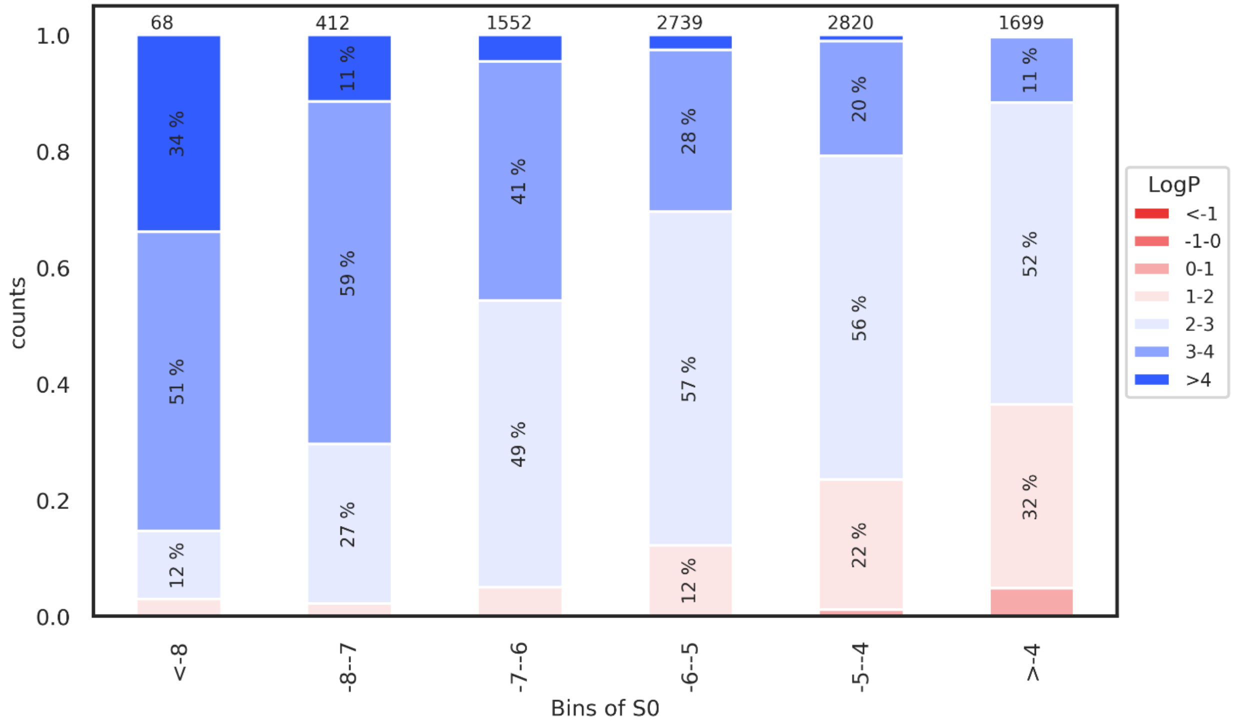

Figure S5. Correlation of intrinsic solubility and logP

7. Molecular representation

Node and edge features can be found in Table S2 and Table S3.

Table S2. Atom attributes used for node representation

Attributes	Explanation
Atom Type	C, N, O, H…
Chirality	CHI_UNSPECIFIED, CHI_TETRAHEDRAL_CW, CHI_TETRAHEDRAL_CCW, CHI_OTHER, misc
Total Degree of the Atom	1 to 10, misc
Formal Charge of the Atom	-5 to 5, misc
Total Number of Hs	0 to 8, misc
Number of Radical Electrons	0 to 4, misc
Hybridization	SP, SP2, SP3, SP3D, SP3D2, misc
Aromatic	False, True
Part of Ring	False, True

Table S3. Bond attributes used for edge representation

Attributes	Explanation
Bond Types	Single, Double, Triple, Aromatic, misc
Stereochemistry of the Bond	STEREONONE, STEREOZ,STEREOE, STEREOCIS, STEREOTRANS, STEREOANY
Part of Conjugation	False, True

8. Model parameters

The model architecture includes two parts: First, a feature extractor composed of GraphGPS and the exponential decay mask. This part is shared across all tasks to extract information from input molecules. The other part is task-specific heads which are connected to the feature extractor and are used to make predictions for individual tasks. For the feature extractor, the number of hidden dimensions is set to 100, with 4 attention heads, 10 message-passing iterations, and a dropout rate of 0.1. Each task-specific head consists of a single layer with the number of hidden dimensions set to 50 and a dropout rate of 0.1. Additional details and implementation specifics can be found in the code repository (https://github.com/JXZhaocc/Muti-task-GT-sol/tree/main).

9. Model performance

Model performance for each of the eight physicochemical properties can be found in Figure S6.

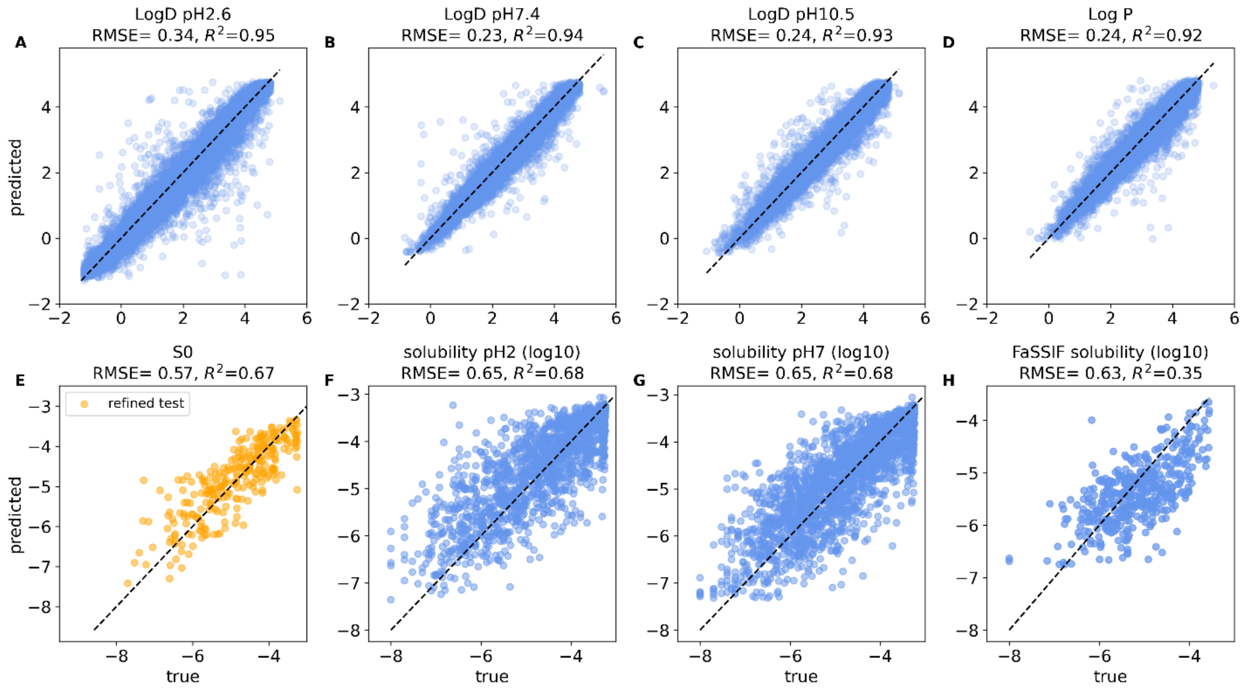

Figure S6. Model performance on test set for the eight physicochemical properties

10. Analysis and reasoning for SC2 data prediction performance

Figure S7 shows the chemical spaces based on latent features obtained from the feature extractor for both J&J and SC2 datasets. The blue region represents the chemical space of the J&J training data, with darker blue indicating areas of higher density and lighter blue representing more sparsely populated regions. Black dots represent compounds from the J&J test data, and the red and green dots represent poorly predicted (|true value - predicted value| > 0.7 log units) and well predicted compounds in the SC2 dataset, respectively. It can be observed from the plot that the poorly predicted compounds were mostly located in the sparsely populated regions of the J&J training data, suggesting that limited representation learned in these regions may contribute to the larger prediction errors. A few poorly predicted SC2 compounds sat in dense regions, which could indicate our multi-task graph transformer model is sensitive to assay shift (assay differences).

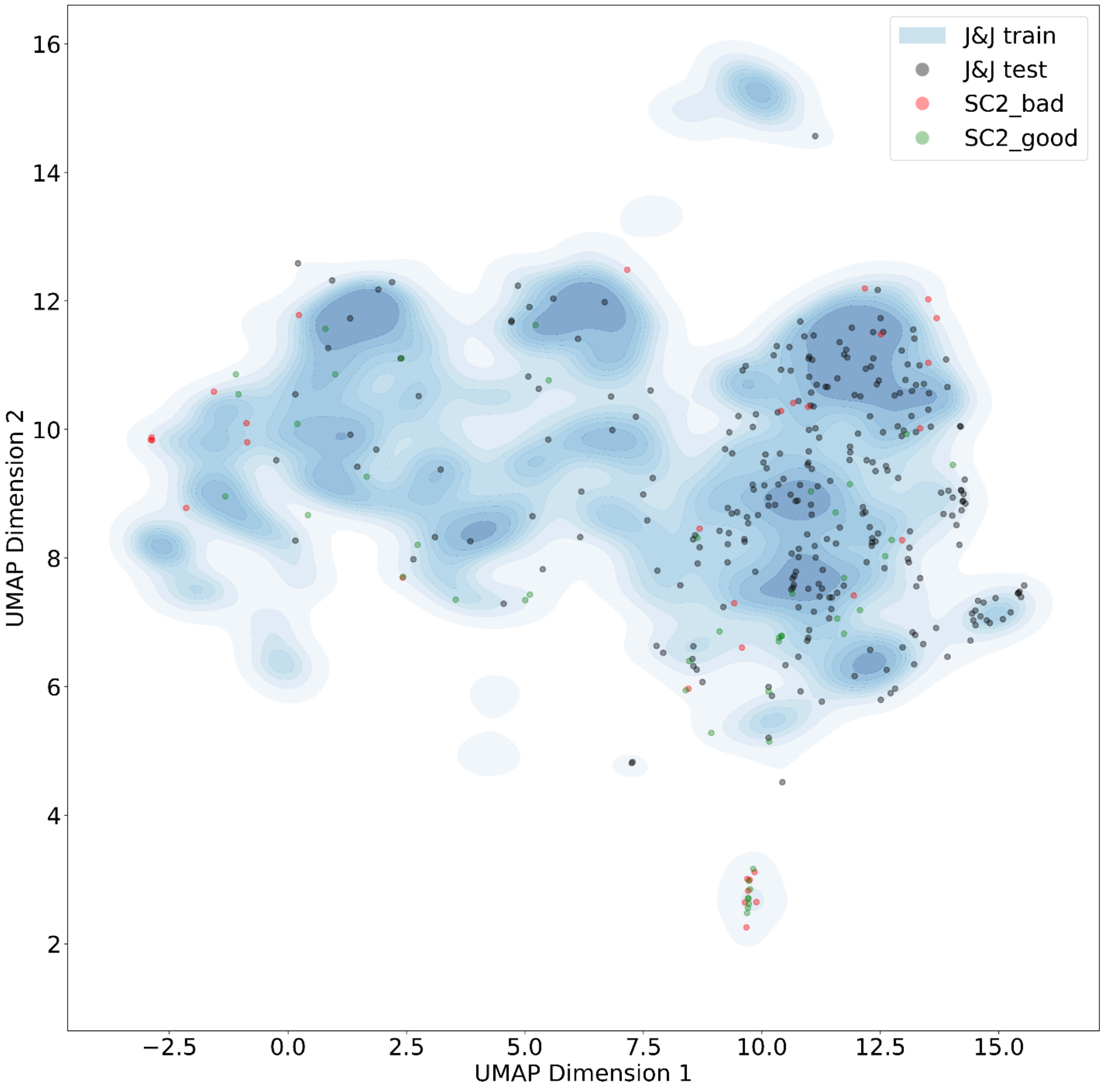

Figure S7. Chemical space based on the latent features for J&J training data, test data, and SC2 data
